# Open and closed states of *Candida antarctica* lipase B: protonation and the mechanism of interfacial activation^1^

**DOI:** 10.1194/jlr.M063388

**Published:** 2015-12

**Authors:** Benjamin Stauch, Stuart J. Fisher, Michele Cianci

**Affiliations:** European Molecular Biology Laboratory-European Bioinformatics Institute (EMBL-EBI),* Wellcome Trust Genome Campus, Hinxton, Cambridge CB10 1SD, United Kingdom; Robinson College,† University of Cambridge, Cambridge CB3 9AN, United Kingdom; Diamond Light Source,§ Didcot, Oxfordshire OX11 0DE, United Kingdom; European Molecular Biology Laboratory (EMBL),** Deutsches Elektronen-Synchrotron (DESY), Hamburg 22607, Germany

**Keywords:** fatty acid/metabolism, lipids/chemistry, enzymology/enzyme regulation, X-ray crystallography

## Abstract

Lipases (EC 3.1.1.3) are ubiquitous hydrolases for the carboxyl ester bond of water-insoluble substrates, such as triacylglycerols, phospholipids, and other insoluble substrates, acting in aqueous as well as in low-water media, thus being of considerable physiological significance with high interest also for their industrial applications. The hydrolysis reaction follows a two-step mechanism, or “interfacial activation,” with adsorption of the enzyme to a heterogeneous interface and subsequent enhancement of the lipolytic activity. Among lipases, *Candida antarctica* lipase B (CALB) has never shown any significant interfacial activation, and a closed conformation of CALB has never been reported, leading to the conclusion that its behavior was due to the absence of a lid regulating the access to the active site. The lid open and closed conformations and their protonation states are observed in the crystal structure of CALB at 0.91 Å resolution. Having the open and closed states at atomic resolution allows relating protonation to the conformation, indicating the role of Asp145 and Lys290 in the conformation alteration. The findings explain the lack of interfacial activation of CALB and offer new elements to elucidate this mechanism, with the consequent implications for the catalytic properties and classification of lipases.

Lipids are, together with proteins and carbohydrates, the basic constituents of the architecture of a living organism. Lipids form membrane systems, which function as physical and chemical barriers separating aqueous compartments, regulating processes such as secretion, endocytosis, and signal transduction. Lipids are one of the major sources of energy, or serve as biological modulators and signal transducers (e.g., steroids, phosphatidylinositol derivatives, etc.) and as vehicles for carrying fat-soluble vitamins. The functional interaction between lipids and proteins is essential for such activities.

Lipases (EC 3.1.1.3) are ubiquitous hydrolases for the carboxyl ester bond of water-insoluble substrates such as triacylglycerols, phospholipids, and other insoluble substrates, acting in aqueous as well as in low-water media. For these reasons lipases are enzymes of considerable physiological significance (1) with high interest also for their industrial applications (2).

The hydrolysis reaction, catalyzed by lipases, does not follow Michaelis-Menten kinetics, and presents a two-step mechanism, defined as interfacial activation, with adsorption of the enzyme to a heterogeneous interface and subsequent enhancement of the lipolytic activity (3). The crystal structures of human pancreatic lipase (4) and of lipase-procolipase complex (5, 6), a fungal enzyme from *Geontrichum candium *(7), and a lipase from *Pseudomonas glumae *(8) showed a lid domain, either a loop or a helix, shielding the active site from the solvent and thus regulating the exposition of the substrate to the catalytic triad. It was postulated that a conformational change would occur in the presence of a lipid-water interface inducing interfacial activation. Thus the presence of a lid domain and interfacial activation have long been used to distinguish lipases from esterases, where the interfacial activation phenomenon was not observed (9). More recently, the observation of a novel lid domain allowed the formulation of a hypothesis for a likely mode of interfacial activation of *Candida antarctica* lipase A (10), while a cutinase from *Trichoderma reesei*, showing interfacial activation behavior and the presence of the lid domain, has been reported as having the kinetic and structural features of a “true lipase” (11).

Among lipases, *Candida antarctica* lipase B (CALB) is the one that found the widest application in many industrial processes because of its high enantioselectivity, wide range of substrates, thermal stability, and stability in organic solvents (12, 13). CALB belongs to the α/β hydrolase fold family with a conserved catalytic triad consisting of Ser, His, and Asp/Glu within an acyl binding pocket and a binding pocket for the moiety of secondary alcohols (14, 15). However, a closed conformation of CALB has never been reported and CALB has never shown any significant interfacial activation (16). Taken together, these observations have led to the conclusion that this behavior was due to the absence of a lid regulating the access to the active site (16).

Whether or not CALB lacks a lid structure is matter of debate, according to several molecular dynamics simulation studies (17–20). CALB has two α-helixes surrounding the active site, namely α5 and α10 (14, 15), which have been shown to be very flexible regions and could work as a lipase lid by a relative motion between them either by increasing the working temperature (19) or by working in organic solvents (17). Such helix motions, in aqueous media, could not be large enough to produce variation in the hydrophobic surface and in the solvent-accessible area or large enough to prevent access to the active site (20). However, the CALB sequence stretch around the α-helix 5 has been shown to significantly influence the catalytic properties of the enzyme, including the enantioselectivity, which, consequently, seems not to be exclusively directed by the fit of the substrate into the enzyme’s active site pocket (18). The issue of the open and closed conformations of CALB is then not only related to the interfacial activation, but it has implications for the catalytic properties of the enzyme (18).

We report here the crystal structure of CALB in open and closed conformation refined at 0.91 Å resolution, representing the highest resolution structure of CALB to date, and of all currently available triacylglycerol lipase X-ray structures (EC 3.1.1.3). Probing the crystals using xenon validated the open and closed conformations. Having available the open and closed conformations, in the same unit cell at atomic resolution, allows relating the protonation states (21, 22) of each monomer to their conformation. Resolving the open and closed conformations offers new explanations for the lack of an interfacial activation mechanism of CALB, pointing to a role of Asp145 and Lys290 in the conformation alteration. A search for homologs with conserved Asp145 and Lys290 residues returns five hits, potentially leading to a new sub-classification of lipases.

## MATERIALS AND METHODS

### Crystallization conditions and xenon derivatization

CALB was purchased from Hampton Research and crystallized without further purification. Crystallization trials were performed at 293 K using the hanging-drop method using a Qiagen™ EasyXtal 15-well plate. One microliter of a 15 mg/ml CALB solution in 20 mM Na(CH_3_COO) (pH 4.8) was diluted with 1 μl of the precipitant solution, made of 200 mM Na(CH_3_COO) (pH 4.8), 20% (w/v) PEG4000, and 10–13% (v/v) 2-propanol. The drop was equilibrated by vapor diffusion against 500 ml of the precipitant solution. Protein crystals of native CALB appeared within 1 week and grew to a size of 0.2 × 0.4 × 0.5 mm^3^ in 3 weeks.

The xenon complex of native CALB was prepared by exposing the crystals to xenon pressure (10 atm) by using the Hampton Research xenon chamber available at the European Molecular Biology Laboratory (EMBL). Xenon incubation times were kept to around 15–20 min. After pressure release, the loop with the crystal was quickly plunged into liquid nitrogen for flash cooling and then transferred to the beam line. The crystal loop was maintained at 100 K with a cold nitrogen stream.

### X-ray diffraction data collection and structure analysis

Diffraction data were collected at 100 K using synchrotron radiation at the EMBL P13 beamline at the Petra III storage ring, Deutsches Elektronen-Synchrotron (DESY), Hamburg (Germany). The beam line was equipped with a Dectris Pilatus 6M detector and a MD2 goniometer (MAATEL-EMBL) with horizontal spindle axis. Crystals were cooled at 100 K with a cold nitrogen stream. The wavelength was set to 0.826 Å, using a Si(III) crystal monochromator (FMB-Oxford). Data were collected from four crystals oriented with different kappa angles while performing helical scans. The data were integrated using the program XDS and scaled with XSCALE (23). Crystals of the native enzyme diffracted to 0.91 Å resolution with unit cell dimensions of a = 39.67 Å, b = 48.94 Å, and c = 71.61 Å and space group P1.

For the xenon complex of native CALB, data collection was carried on at a wavelength of 2.25 Å, chosen to enhance the anomalous signal from the expected xenon atoms and warrant the necessary resolution for the experiments. At λ = 2.25 Å, close to the L_I_ edge of Xe, the imaginary component of the anomalous dispersion f” is of 13.14 e−. Crystals of the xenon complex diffracted to 2.27 Å resolution with unit cell dimensions of a = 39.6 Å, b = 48.8 Å, and c = 71.6 Å and space group P1. The resolution of data for the xenon complex was limited by the geometry of the camera.

The structures of native CALB and of the xenon complex were solved by molecular replacement using MOLREP (24) starting from the deposited structure of CALB as search model [Protein Data Bank (PDB): 1TCA (14)]. The models were subjected to rigid-body minimization and subsequently to refinement steps with REFMAC (25). For the native structure, anisotropic temperature factors refinement was used and all the hydrogen generated, with automatic weighting scheme, given the value of 53 for ratio reflection/atom. For the xenon complex, isotropic temperature factors refinement was used. Manual rebuilding of the models were performed using the COOT graphic interface (26) by inspecting the electron density map, calculated with 2F_obs_ − F_calc_ or F_obs_ − F_calc_ coefficients and calculated phases from the model. Models were validated using the PDB_REDO web server (27). The native structure and its xenon complex were refined to an R_factor_ and R_free_ (5% of data) of 11.1 and 13.2%, and of 13.9 and 19.4%, respectively. Data collection and refinement statistics are reported in **Table 1**. The protonation-state determination of the amino acid side chains followed the protocol described by Fisher et al. (22).

**TABLE 1.** Data collection, processing, and refinement statistics for the native *Candida antarctica* and xenon complex

	Native	Xenon Complex
Data collection		
Wavelength (Å)	0.826	2.250
Detector	Pilatus 6M	Pilatus 6M
Number of crystals used	4	2
Space group	P1	P1
Unit cell (a, b, c, Å)	39.67, 48.94, 71.61	39.66, 48.89, 71.68
Unit cell (α, β, γ, °)	88.73, 97.15, 108.44	87.79, 98.07, 108.17
Resolution range (Å)*^a^*	71.04–0.91 (0.93–0.91)	70.96–2.27 (2.36–2.27)
Total number of reflections*^a^*	5,925,539 (74,630)	133,177 (7,287)
Unique reflections*^a^*	343,684 (20,871)	21,330 (1,559)
Multiplicity*^a^*	17.2 (3.5)	6.2 (4.6)
Completeness*^a^* (%)	94.5 (77.4)	92.7 (70.5)
R_sym_*^a^*,*^b^* (%)	10.9 (56.7)	5.7 (34.0)
Mean (I) half-set correlation CC(1/2)	99.9 (80.2)	99.9 (95.0)
Mean I/σ(I)*^a^*	13.65 (1.59)	29.5 (7.2)
Refinement statistics		
Number of monomers in the asymmetric unit	2	2
R_factor_*^c^* (%)	11.1	13.9
R_free_*^c^* (%)	13.2	19.4
Cruickshank’s DPI for coordinate error*^d^* based on R_factor_ (Å)	0.01	0.23
Wilson plot B-factor	8.69	13.78
Average all atom B-factor*^e^*	13.0	14.4
RMS (bonds)	0.02	0.007
RMS (angles)	2.23	1.12
Total number of nonhydrogen atoms*^c^*	5,955	5,193
Total number of water molecules	1,019	484
Solvent content (%)	38.0	
Matthews coefficient (Å^3^/Da)	1.98	
Ramachandran plot*^f^*		
Most favored region (%)	90.2	90.2
Additionally allowed region (%)	9.1	9.1
Generously allowed region (%)	0.8	0.8
Disallowed region (%)	0.0	0.0

a Highest resolution bin in parentheses.

b R_sym_ = Σ_hkl_ Σ_j_|I_j_ − <I>|/Σ_hkl_ Σ_j_ I_j_ where I is the intensity of a reflection, and <I> is the mean intensity of all symmetry related reflections j.

c Taken from REFMAC5 (25) (R_free_ is calculated using 5% of the total reflections that were randomly selected and excluded from refinement).

d DPI = sqrt(N_atoms_/[N_refl_-N_params_)]R_factor_ D_max_ compl^−1/3^, where N_atoms_ is the number of the atoms included in the refinement, N_refl_ is the number of the reflections included in the refinement, D_max_ is the maximum resolution of reflections included in the refinement, compl is the completeness of the observed data, and for isotropic refinement, N_params_ ≈ 4N_atoms_ (31).

e Taken from BAVERAGE (28).

f Taken from PROCHECK (30).

## RESULTS

### Overall structure

**Fig. 1.**
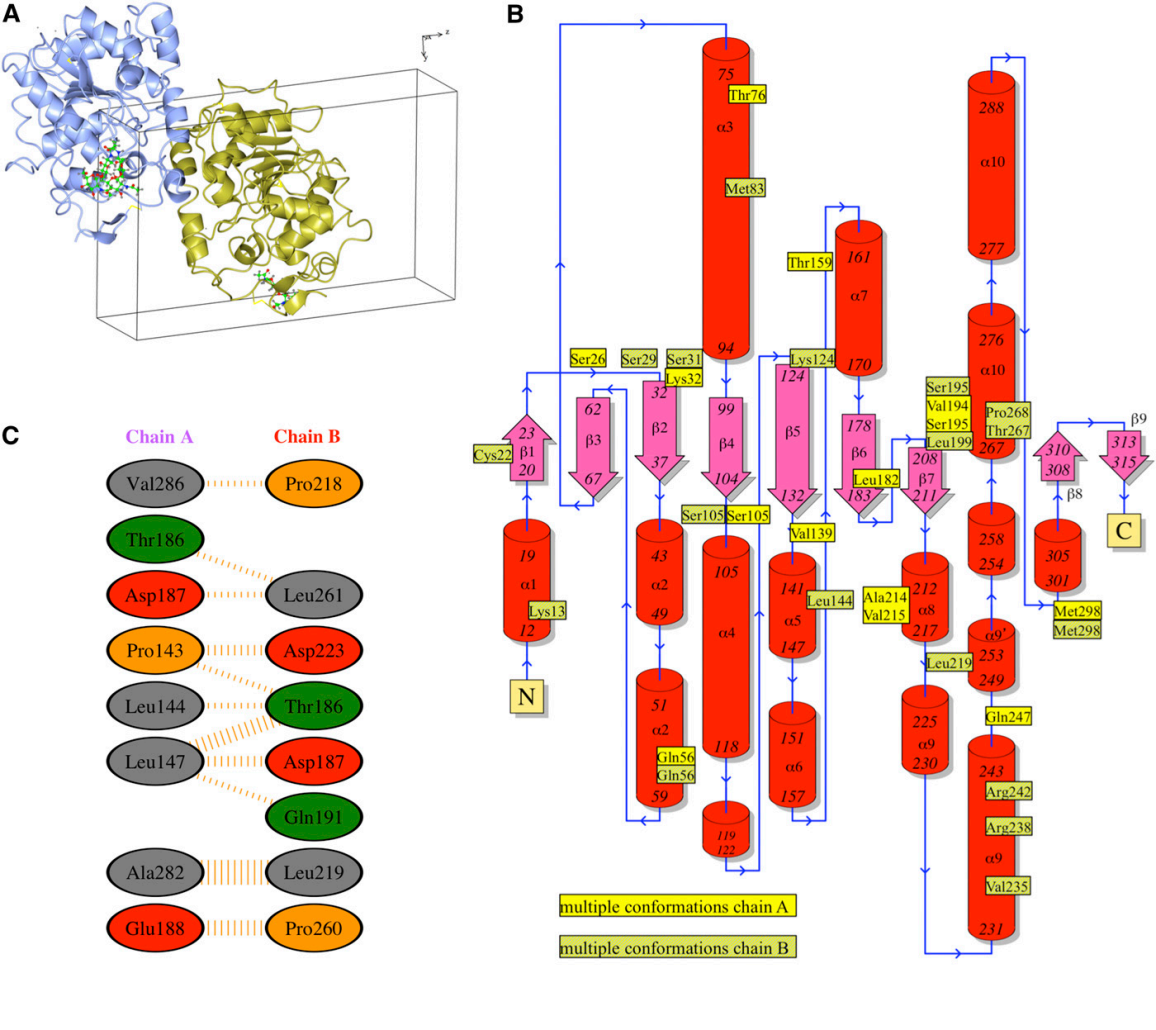
Graphical representation of CALB. A: Asymmetric unit. B: General secondary structure topology with the position of residues showing multiple conformations highlighted. C: Nonbonded contacts between residues, the width of the striped line is proportional to the number of atomic contacts. Figures were generated using PDBsum web server (40).

In the asymmetric unit there are two monomers (**Fig. 1A**), which show the α/β type fold described by Uppenberg et al. (14) (Fig. 1B). These two monomers interact via a network of nonbonded contacts between residues Pro143, Leu144, Leu147 (α-helix 5), Thr186, Asp187, Glu188 (loop between sheet β6−β7), Ala282, and Val286 (α-helix 10) in monomer A and Thr186, Asp187, Gln191 (loop between sheet β6-β7), Pro218, Leu219, Asp223 (α-helix 5 and subsequent loop), Pro260, and Leu261 (loop between α-helix 9 and 10) in monomer B, representing interface areas of 463 and 442 Å^2^ in monomer A and B, respectively (Fig. 1C). Two *N*-acetylglucosamine molecules are observed in both monomer A and monomer B. In monomer A, the triad Asn74-NAG-NAG shows two alternative conformations, while in monomer B only a single conformation is observed. Several multiple amino acid side chain conformations are observed throughout the structure (Fig. 1B).

### Open and closed conformations

**Fig. 2.**
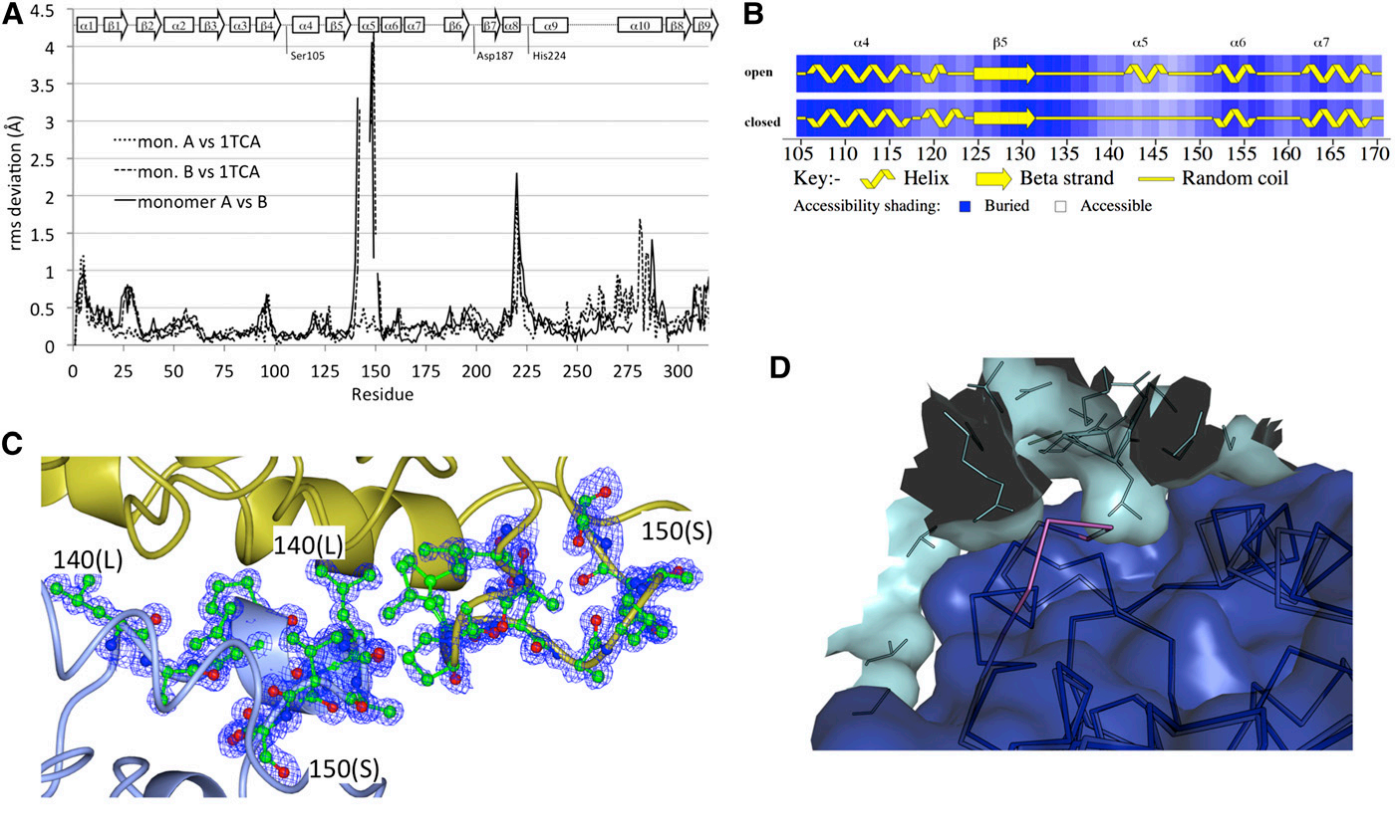
Structural evidence of open and closed conformation in CALB. A: The rms deviation values of Cα atoms for monomers A and B computed against the currently deposited CALB structure [PDB code 1TCA (14)] versus residue number. B: PROCHECK (30) analysis of the secondary structure region surrounding α-helix 5, showing the unfolding of residues 140–147. C: Ribbon representation of the residue range 140–147 (monomer A, folded in light blue color; monomer B, defolded in gold color) superimposed to the F_o_ − F_c_ difference Fourier OMIT map (blue mesh; contour level 3.0σ, calculated without the residues) at 0.91 Å resolution. D: Steric clash between extended closed lid segment/α-helix and neighboring monomers in other crystal forms of CALB. The structure of CALB assuming the closed lid conformation described in this study (magenta, shown as backbone trace/ribbon) was superimposed to the structure 1TCA (blue ribbon and surface) present in an open conformation. Residue Pro143 (magenta) in the closed conformation would sterically clash with residue Leu199 of a neighboring CALB monomer (cyan) within the same crystallographic unit cell. The superimposition was carried out and images were generated using PyMol (41).

The root-mean-square (rms) deviations of the Cα atoms of CALB monomers A and B, reported here, against the monomer A of the structure reported by Uppenberg et al. (14) (PDB code 1TCA) are 0.34 Å and 0.54 Å, respectively, as calculated using SUPERPOSE (28) over 310 residues (**Fig. 2A**). The rms deviation values of Cα atoms of monomer A against monomer B, presented here, are 0.53 Å. The rms deviation values (Fig. 2A) indicate that the secondary structure of monomer A is maintained and consistent with the classical open conformation previously observed (14, 15, 29), while the rms deviation values of Cα atoms for monomer B show a dramatic change between residues 140 and 147. The residue range Leu140-Ala141-Gly142-Pro143-Leu144-Asp145-Ala146-Leu147, corresponding to α-helix 5, undergoes a conformational change from an α-helix structure to a loop, as clearly shown by the assignment of the secondary structure computed with PROCHECK (30) (Fig. 2B). The electron density maps at 0.91 Å resolution clearly describe the residue range 140–147 of monomer A as an α-helix, corresponding to an open conformation, and of monomer B as an unfolded loop, corresponding to a closed conformation (Fig. 2C). The residue range 140–147 presents Asp145 as the only amino acid with a charged side chain.

**Fig. 3.**
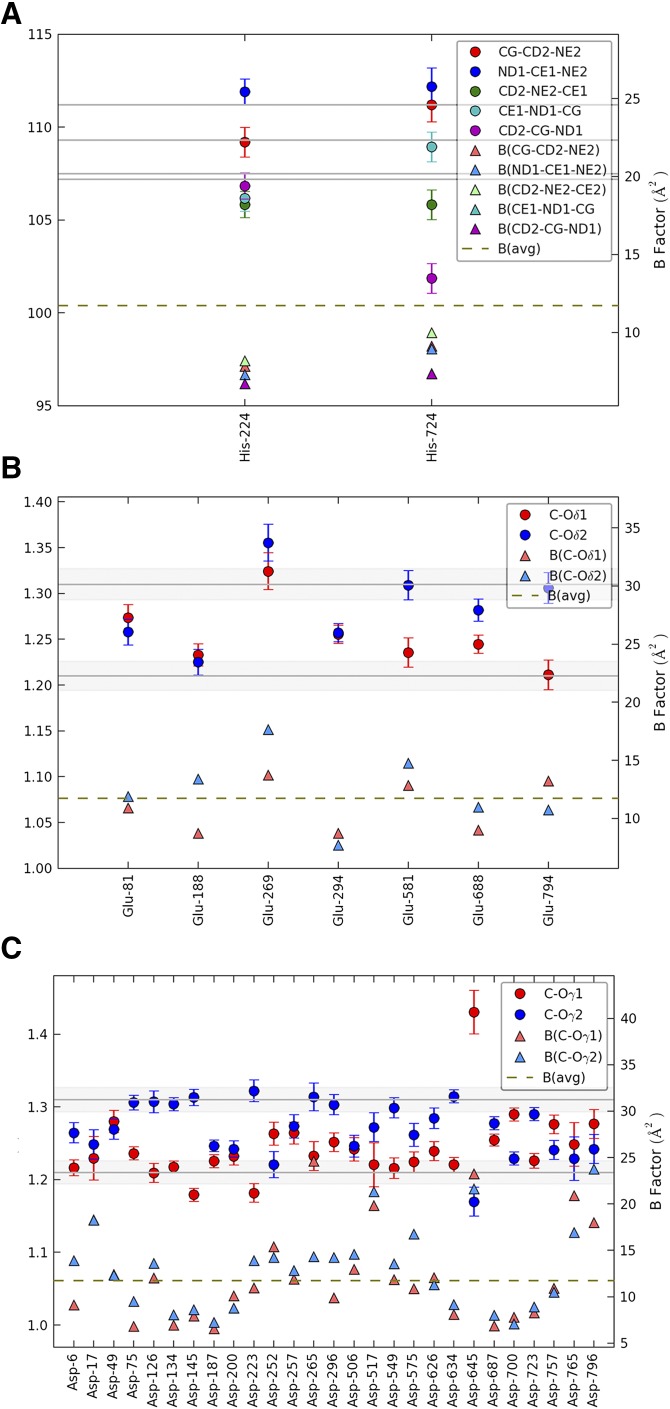
Bond length and bond angle analysis. Histidine (bond angles) (A); glutamic acid (bond lengths) (B); aspartic acid residues (bond lengths) (C). Values are indicated with standard deviations.

**Fig. 4.**
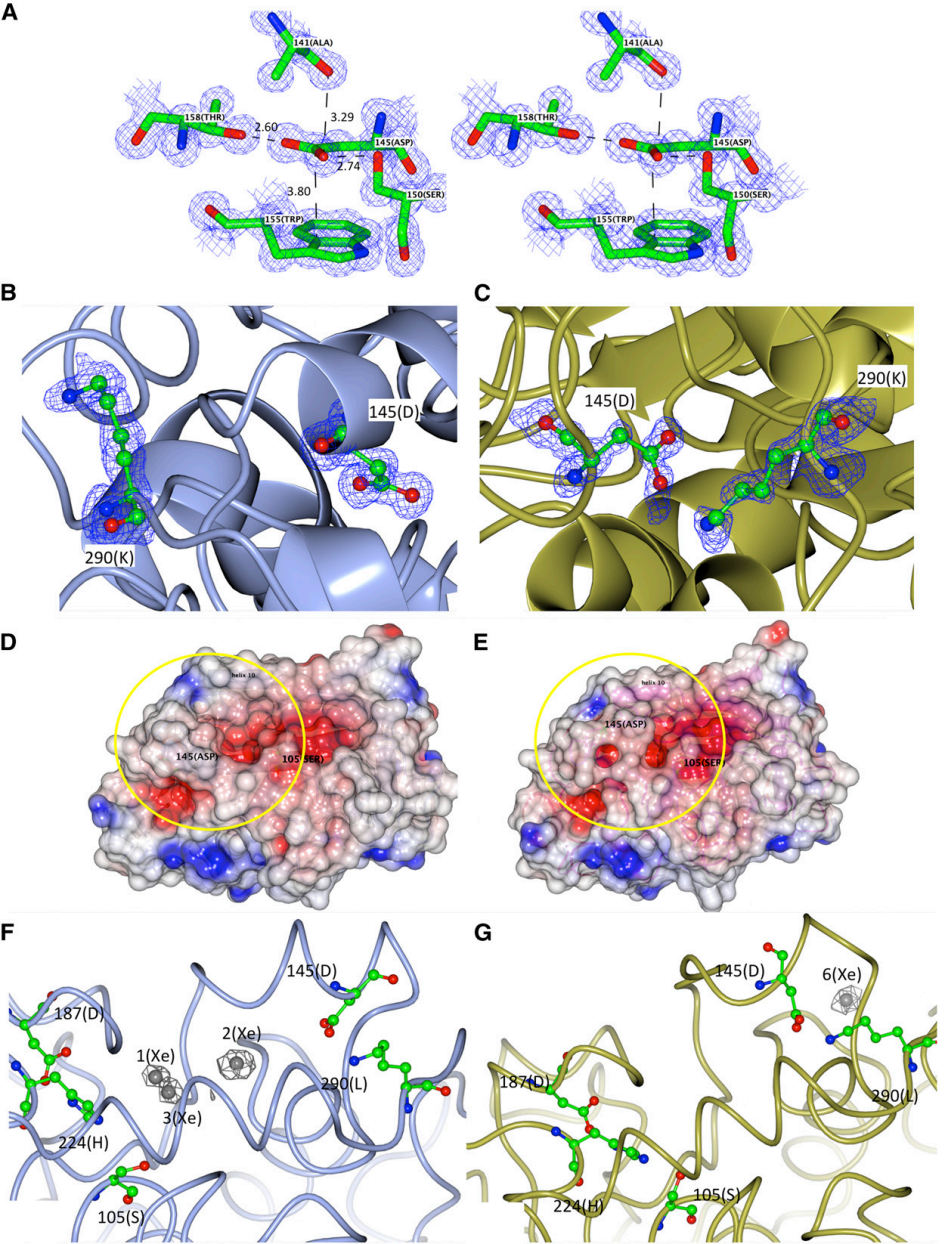
Open and closed conformations of CALB. Amino acid residues are colored as carbon, green; oxygen, red; nitrogen, blue. A: Stereoview of the Asp145 region in the open conformation with 2F_o_ − F_c_ electron density map shown contoured at 1.5σ level. B, C: The amino acid residues Asp145 and Lys290 in monomer A (light blue) open conformation (B) and monomer B (gold) closed conformation (C), superimposed to the F_o_ − F_c_ difference Fourier OMIT map (blue mesh; contour level 3.0σ, calculated without the residues). D, E: Representation of CALB surface in open conformation (D) and closed conformation (E) with the Leu140-Ile147 region highlighted. Positive electrostatic potential regions are depicted in blue, negative electrostatic potential regions are depicted in red. White is neutral. F, G: Xenon sites (represented as spheres in gray) superimposed to the anomalous difference Fourier map (green mesh; contour level 4.0σ, phases calculated omitting the xenon atoms) present into the active site in open conformation (F) and absent in the cavity in closed conformation (G). Images were generated using CCP4MG (42).

The final model of CALB has a standard uncertainty for the positional parameters of all atoms of 0.015 Å, as calculated using Cruickshanck’s dispersion precision indicator (**DPI**) factor (31) (Table 1), which is six times lower than the values of the best currently reported CALB structures. Determination of the protonation state by bond length analysis (21, 22) of the Asp and Glu amino acid side chains (**Fig. 3**) resulted in a final computed average standard deviation of 0.005 Å. The analysis reveals that, in chain A, the carboxylic group of Asp145 is protonated with bond lengths of C-Oγ2 ≈ 1.3 Å and of C-Oγ1 ≈ 1.2 Å, while in chain B it is deprotonated due to salt bridge formation, with bond lengths of the Asp145 carboxylic group of C-Oγ2 < 1.2 Å and of C-Oγ1 > 1.4 Å outside standard values. In the open conformation, Asp145 is placed at a distance of 3.8 Å above the indole group of Trp155, within hydrogen bond distance from the carbonyl group of the main chain of Ala141, the hydroxyl group of Ser150, and Thr158 side chains (**Fig. 4A**). The side chain of Asp145 shifts by 7.4 Å, reaching the side chain of Lys290 at a distance of 2.7 Å measured between Lys290 NZ atom and Asp145 Oγ1 atom, thus generating a salt bridge (Fig. 4B, C). The consequent large conformational change of the Leu140-Leu147 region results in the closing of the CALB active site, as shown by the space-fill representation (Fig. 4D, E).

Pressurizing the crystal with xenon probed the tightness of the closed lipase conformation. Three xenon atoms, with anomalous signal bigger than 4σ, were located in monomer A in the catalytic site in close proximity to the catalytic triad. In particular, the three xenon atoms (8.3σ, 6.3σ, and 6σ map level, respectively, refined with fractional occupancies) in monomer A are located above His224, in the hydrophobic region of the catalytic site defined by Leu144, Val154, Ile189, Leu219, Val221, Ala281, Ala282, Ile285, and Leu287 (Fig. 4F). No xenon atoms were observed in the active site of monomer B, thus confirming that the conformational rearrangement of the residue range 140–147 prevents the access of any substrate to the active site (Fig. 4G). Xenon is also found in the hydrophobic cavity defined by Met83, Val101, Leu115, Ile121, Val125, Val37, and Ile187 in both monomer A (10.2σ) and monomer B (10.5σ). A further site is found next to Trp155 in monomer B (7.2σ).

The rms deviation values of Cα atoms (Fig. 2A) confirm a reorientation of the loop residues between α-helixes 8 and 9, localized on Leu219 and around α-helix 10. These shifts are compatible with the protein-protein interaction and with the crystal packing. In particular, Leu219 of monomer A, in open conformation, is pointing toward α-helix 10 of the symmetry-related molecule in closed conformation, in a proximal position to Lys290. Leu219 of monomer B is found above the active site of monomer A.

### Active sites

In the active sites of both monomer A and monomer B, Ser105 is refined with multiple conformations, with the hydroxyl group swinging toward and away from NE2 of His224 and one water molecule. In both active sites, molecules of isopropanol are found tethered by the hydrogen bonding with the hydroxyl group of the side chain of Thr40, with the alkane chain positioned in the void between Asp134, Gln157, and Ile189. A network of waters is found within the active site and extending from the catalytic triad toward α-helix 5 and α-helix 10.

**Fig. 5.**
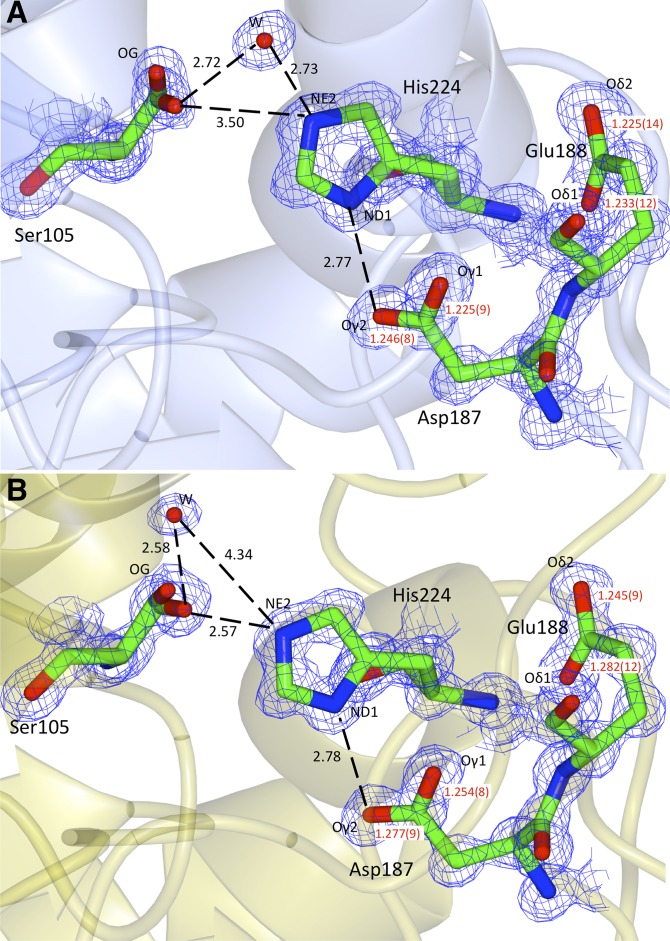
Active site of CALB. A 2F_o_ − F_c_ electron density map shown contoured at 1.5σ level: open conformation (A); closed conformation (B). Bond lengths in red and hydrogen bond distances in black are reported in ångström.

The bond length analysis (22) shows that, in monomer A (**Fig. 5A**), the side chains of Asp187 and Glu188 are deprotonated, with bond lengths of C-Oγ1 = 1.225(9) Å and C-Oγ2 = 1.246(8) Å for the Asp187 residue and of C-Oδ1 = 1.233(12) Å and C-Oδ2 = 1.225(14) Å for the Glu188. The protonation state in His224 can be rationalized by analyzing the chemical environment as suggested by Fisher et al. (22). In monomer A, Asp187 forms hydrogen bond via Oγ2 with the His224 ND1 proton (2.79 Å), while a water molecule donates protons to NE2 (2.73 Å). In monomer B (Fig. 5B), the side chain of Asp187 is also deprotonated with bond lengths of C-Oγ1 = 1.277(9) Å and C-Oγ2 = 1.254(8) Å. The side chain of Glu188 appears to be protonated or partially protonated, as evident from the different C-Oδ1 and C-Oδ2 distances, where the C-Oδ1 bond, hydrogen bonded to the N-H of the backbone of His224, is 0.05 Å shorter than the C-Oδ2 facing toward a water molecule. When looking at the maps and the structures of His224, ND1 proton makes a hydrogen bond to the deprotonated Asp187. Ser105 proton hydrogen bonds to His224 NE2. In both monomer A and monomer B, a water molecule is shared between His224 and Ser105 (Fig. 5A, B).

## DISCUSSION

**Fig. 6.**
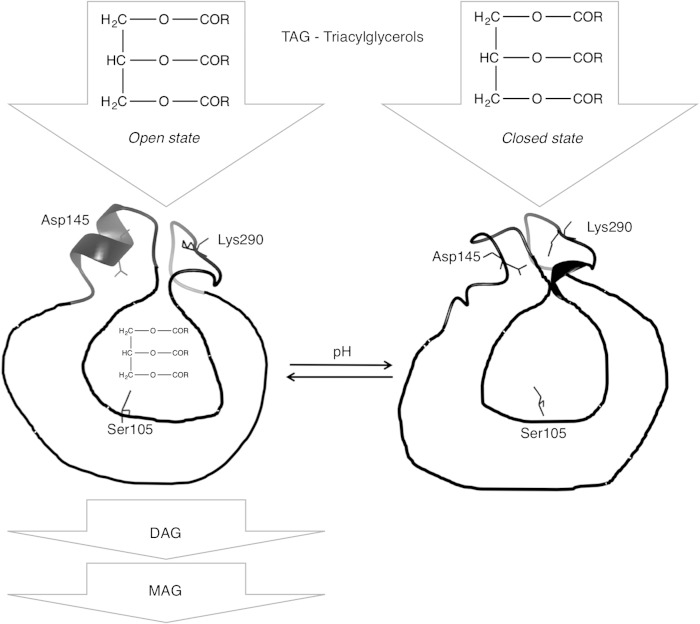
Diagrammatic summary of the overall mechanism of CALB catalysis. A triacylglycerol molecule (TAG) is hydrolyzed to diacylglycerol (DAG) and monoacylglycerol (MAG) when CALB is present in open state.

The presence of a lid covering the active site in CALB has long been a matter of debate. The lack of structural evidence (14, 15) and the lack of obvious interfacial activation during catalysis (16) have been the main argument against the existence of a lid in CALB. The α-helix 5 and α-helix 10 are shown here to be responsible for the closed conformation of CALB, and describe a completely unforeseen closing mechanism (**Fig. 6**) at atomic level. In the open conformation, α-helix 5 shows a series of aliphatic residues that line the channel leading to the active site (14) with the only polar residue, Asp145, making hydrogen bonds with the side chains of Ser150 and Thr158. When closing the active site, the α-helix 5 undergoes a dramatic conformational change to an unstructured loop while bringing the carboxylic group of Asp145 close to the side chain of Lys290, thus forming a salt bridge. The spatial rearrangement of Lys290 brings α-helix 10 closer to new lid region, completing the closure of the catalytic cavity. The unfolding of α-helix 5 results in the closing of the lid over the cavity, preventing the access of any substrate to the catalytic site, as shown by probing binding sites with xenon.

Xenon is able to bind to a protein in closed intramolecular hydrophobic cavities, accessible active sites, intermolecular cavities, and channel pores, as the result of noncovalent weak-energy van der Waals forces (32), by diffusing rapidly toward potential interaction sites through the solvent channels of a protein crystal (32). The relatively high atomic number and its anomalous dispersion of xenon, together with data collection at long wavelengths, make it possible to locate it within protein structures in crystallography (33).

The location of three xenon atoms in monomer A, in close proximity to the catalytic triad, and none in monomer B, confirms that the conformational rearrangement of α-helix 5 seals the active site. In our structure the lid region in monomer B is held in place by a close contact with the α-helix 9’ region of the symmetry related monomer A. No evident hydrogen bond pattern is present, and the interaction happens via hydrophobic surface patches from each monomer.

Comparison with previously reported CALB structures reveals reasons for the absence, so far, of structural evidence for this closed lid conformation. In the structures reported by Uppenberg et al. (14) (PDB identifiers 1TCA, 1TCB, and 1TCC), the range Asp145-Pro143 would clash with residue Leu199 of a symmetry-related CALB monomer that is part of a face-to-face (i.e., binding site to binding site) dimer, if the lid segment would adopt the more extended closed conformation (Fig. 2D). Instead, in these structures, the lid segment adopts the more compact open α-helical conformation. In our structure, however, the monomers are shifted with respect to each other such that for the monomer in closed conformation, this clash does not occur (Fig. 2C). A similar clash between residues Asp145-Pro143 in one monomer and Leu199 in the other monomer would occur for a closed lid conformation in an additional CALB structure [PDB identifier 1LBS (15)] where the open conformation of α-helix 5 has been modeled (15). Interestingly, in the same study, the authors obtain an alternative crystal form of CALB in the presence of Tween 80 at pH 4.0 that is unusually loosely packed and has a solvent content of over 60% (15). The hydrophobic surface around the active site is fully exposed to the solvent, and the lid segment is found in the open conformation. The absence of the closed conformation, in this case, cannot be attributed to steric reasons alone, as the crystal packing would allow for the lid to adopt the more extended closed conformation. However, it is tempting to speculate that the predominantly hydrophobic lid segment, α-helix 5, upon exposure to polar solvents and/or high pH, prefers a more compact helical conformation, minimizing unfavorable solvent contacts. In our structure, however, the lid segment is better protected against solvent exposure by tighter crystal packing (solvent content 38%).

CALB was crystallized in conditions very similar to those reported by Uppenberg et al. (29). We buffered the precipitation solution to pH 4.8, a value that is just above the pK_a_ of the side chains Asp (pK_a_ = 3.86) and Glu (pK_a_ = 4.07) residues. At pH 4.8, it is reasonable to expect that most of the carboxylic groups would be in the deprotonated state, subject to the local protein environments.

Figure 3, reporting the bond lengths of C-Oγ1 and C-Oγ2 of the carboxylic group of aspartic residues, and of C-Oδ1 and C-Oδ2 of the carboxylic group of glutamic residues, clearly shows that most Glu and Asp residues are, in fact, in a deprotonated state, but exceptions in a protonated state are also present. However, the protonation state of the charged residues in the active site of the two monomers (**Table 2**) indicates that the charge inside the cavity is conserved. Residue Asp187 maintains its deprotonated state, Glu188 is deprotonated in monomer A and protonated in monomer B, while Asp145 is protonated in monomer A and deprotonated in monomer B (Table 2) as it forms a salt bridge.

**TABLE 2.** Protonation states of the charged residues in the active site of the two monomers

Residue	Monomer A (Open Conformation)		Monomer B (Closed Conformation)	
	Protonation State	Charge	Protonation state	Charge
Asp134	Protonated	0	Protonated	0
Asp145	Protonated	0	Deprotonated*^a^*	−1
Asp187	Deprotonated	−1	Deprotonated	−1
Glu188	Deprotonated	−1	Protonated	0
His224	Single protonated	0	Single protonated	0
	Overall charge	−2	Overall charge	−2

a In ionic pair with the side chain of Lys290.

The bond lengths of Asp187 within the catalytic triad in open and closed conformations are consistent with the bond lengths of Asp102 in trypsin and of Asp32 in subtilisin reported by Fisher et al. (22), and further confirm previous NMR (34) and crystallographic evidence (35) that the key catalytic proton is positioned between the two key catalytic residues, His and Asp, rather than being fixed on the histidine, thus supporting the theory that the proton may, in fact, be involved in a low-barrier hydrogen bond (34).

Overall there is no change of the net charge on the protein in the open and closed conformations (Table 2) following the destructuring of α-helix 5 and the subsequent ion pairing of Asp145 with Lys290 from α-helix 10.

The optimal pH for catalysis is 7, and the enzyme is stable in aqueous media in the range of pH 3.5–9.5 (12), values close to the pK_a_ of the side chains of Asp145 and Lys290 residues discussed before. It was already noted that, in the monoclinic form P2_1_, raising the pH from 3.6 to 5.5 would cause dramatic changes to α-helix 10, whose residues would change from an ordered structure to a disordered structure, manifested by a lack of continuous electron density (14). The lid region itself, with its primary (amino acid composition and number of residues) and secondary structure, can modulate the “interfacial activation behavior” of CALB, but also of lipases in general. Chimeras of CALB, generated by lid swapping, displayed large variations in kinetic constants and enantiomeric ratio for hydrolysis of p-nitrophenyl esters (18). So, while the previous studies pointed toward an activation of lipases based on an interfacial oil-water mechanism, in the case of CALB, the environmental pH could also play a role. At pH 7, CALB in solution is likely to oscillate between the open and the closed conformation; hence, the reason for the lack of interfacial activation of CALB must be searched for elsewhere, rather than in the lack of a lid region in the structure. It is noteworthy that the removal of the Asp145 from the α-helix 5 region in the chimeras of CALB removed the activity pH dependence (18), clearly indicating a relationship between optimal activity pH and primary sequence of the lid region. The two variants, where the lid region was cloned from *Neurospora crassa* and from *Gibberella zeae*, on the other hand, yielded surprisingly low specific activities in the assay, with no clear pH dependency (18). In these two variants, the Asp145 is missing from the α-helix 5 sequence.

**Fig. 7.**
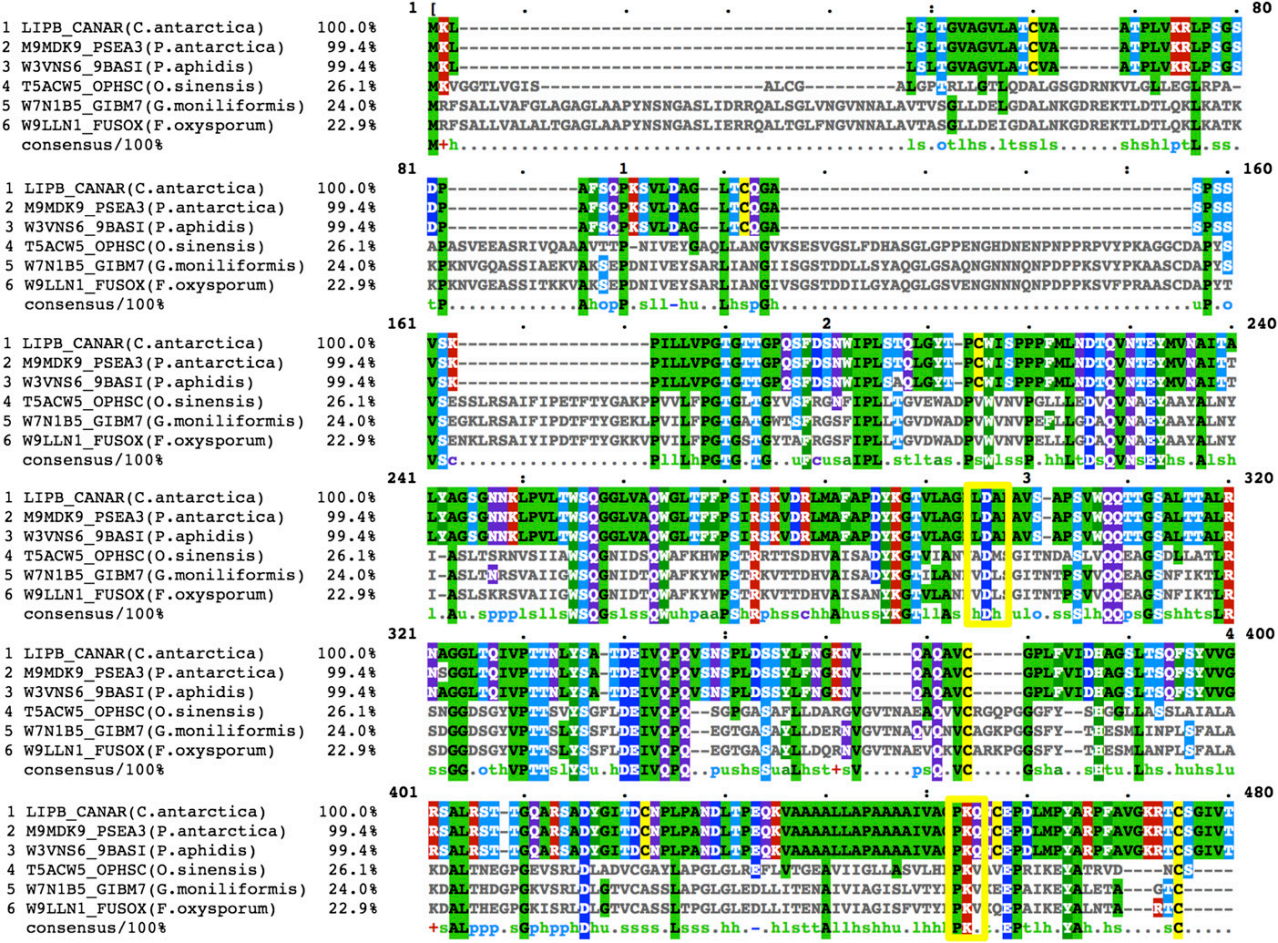
Alignment of CALB homologs with conserved Asp145 and Lys290 residues. UniProt gene codes, organism, and identity (percent) are reported from the left, respectively. Yellow boxes mark the position of Asp145 and Lys290 residues. Amino acid coloring scheme is according to the amino acid family.

A BlastP search (36) using CALB sequence as a search motif returns more than 50 results with sequence identity higher than 20% (results not shown). However, among these, only five hits are found, which conserve Asp145 in the α-helix 5 region and Lys290 after the α-helix 10 region (**Fig. 7**). *Candida antarctica* (LIPB_CANAR), *Pseudozyma antarctica* (M9MDK9_PSEA3), and *Pseudozyma aphidis* (W3VNS6_9BASI) share a sequence identity of more than 99%, while *Ophiocordyceps sinensis* (T5ACW5_OPHSC), *Gibberella moniliformis* (W7N1B5_GIBM7), and *Fusarium oxysporum* (W9LLN1_FUSOX) have a sequence identity of about 25% with *Candida antarctica* (LIPB_CANAR). The fact that Asp145 is rarely conserved in the sequence of lipases, would explain why, among lipases, CALB is the only lipase reported so far, which does not show the interfacial activation behavior typical of other lipases, but rather shows a “pH activation” behavior, as shown by Skjøt et al. (18). Our structure has shown that a salt bridge is formed between Asp145 and Lys290 upon lid closure. In the absence of Asp145, such a salt bridge cannot form, thus a pH-dependent behavior is not possible. Because of the presence of Asp145 in the α-helix 5 region, CALB does represent a class of its own among lipases on the basis of the sequence homology and its characteristic behavior as lipase.

Several molecular dynamics simulation studies (17–20) have shown that α-helix 5 is a highly dynamic region and works as a lipase lid depending on the working temperature (19) or the working organic solvents (17). Derewenda et al. (37) suggested that the interfacial activation has both enzyme and substrate components, and that conformational changes in the enzyme are essential, but not sufficient, for its maximum activity. Schrag et al. (38) proposed that the open or closed conformation of lipases was determined by the solution conditions, and perhaps the dielectric constants, on the basis of the crystallization conditions of various microbial lipases and their observed conformations. Vice versa, in nonaqueous media, the lack of interfacial activation suggested that the lid was predominantly closed, thus resulting in reduced enzymatic activity (39).

Here we have shown that the basis of the interfacial activation for lipases should not, therefore, be discussed on the basis of the presence or the absence of a lid region in an enzyme structure, which could go unobserved, but should rather be related to both the amino acid composition of the lid regions and how and why these regions react to changes in the dielectric value or ionic strength of the media. The interfacial activation phenomenon is then the convolution of the properties of the substrate, the media, and the enzyme with its fold and with its amino acid sequence.

## References

[1] Wong H. 2002 The lipase gene family. J. Lipid Res. 43: 993–999. 12091482 10.1194/jlr.r200007-jlr200

[2] Lotti M., and Alberghina L. 2007 Lipases: molecular structure and function. *In* Industrial Enzymes: Structure, Function and Applications. J. Polaina and A. P. MacCabe, editors. Springer, Dordrecht, The Netherlands. 263–281.

[3] Sarda L., and Desnuelle P. 1958 Actions of pancreatic lipase on esters in emulsions. Biochim. Biophys. Acta. 30: 513–521. 13618257 10.1016/0006-3002(58)90097-0

[4] Winkler F. K., D’Arcy A., and Hunziker W. 1990 Structure of human pancreatic lipase. Nature. 343: 771–774. 2106079 10.1038/343771a0

[5] van Tilbeurgh H., Sarda L., Verger R., and Cambillau C. 1992 Structure of the pancreatic lipase-procolipase complex. Nature. 359: 159–162. 1522902 10.1038/359159a0

[6] Egloff M-P., Marguet F., Buono G., Verger R., Cambillau C., and Tilbeurgh H. v. 1995 The 2.46 A resolution structure of the pancreatic lipase-colipase complex inhibited by a C11 alkyl phosphonate. Biochemistry. 34: 2751–2762. 7893686 10.1021/bi00009a003

[7] Schrag J. D., and Cygler M. 1993 1.8 Å refined structure of the lipase from Geotrichum candium. J. Mol. Biol. 230: 575–591. 8464065 10.1006/jmbi.1993.1171

[8] Noble M. E. M., Cleasby A., Johnson L. N., Egmondb M. R., and Frenkenb L. G. J. 1993 The crystal structure of triacylglycerol lipase from Pseudomonas glumae reveals a partially redundant catalytic aspartate. FEBS Lett. 331: 123–128. 8405390 10.1016/0014-5793(93)80310-q

[9] Verger R. 1997 “Interfacial activation” of lipases: facts and artifacts. Trends Biotechnol. 15: 32–38.

[10] Ericsson D. J., Kasrayan A., Johansson P., Bergfors T., Sandstrom A. G., Backvall J. E., and Mowbray S. L. 2008 X-ray structure of Candida antarctica lipase A shows a novel lid structure and a likely mode of interfacial activation. J. Mol. Biol. 376: 109–119. 18155238 10.1016/j.jmb.2007.10.079

[11] Roussel A., Amara S., Nyyssola A., Mateos-Diaz E., Blangy S., Kontkanen H., Westerholm-Parvinen A., Carriere F., and Cambillau C. 2014 A cutinase from Trichoderma reesei with a lid-covered active site and kinetic properties of true lipases. J. Mol. Biol. 426: 3757–3772. 25219509 10.1016/j.jmb.2014.09.003

[12] Anderson E. M., Larsson K. M., and Kirk O. 1998 One biocatalyst—many applications: the use of Candida antarctica B-lipase in organic synthesis. Biocatal. Biotransformation. 16: 181–204.

[13] Rotticci D., Rotticci-Mulder J. C., Denman S., Norin T. r., and Hult K. 2001 Improved enantioselectivity of a lipase by rational protein engineering. ChemBioChem. 2: 766–770. 11948859 10.1002/1439-7633(20011001)2:10<766::AID-CBIC766>3.0.CO;2-K

[14] Uppenberg J., Hansen M. T., Patkar S., and Jones T. A. 1994 The sequence, crystal structure determination and refinement of two crystal forms of lipase B from Candida antarctica. Structure. 2: 293–308. 8087556 10.1016/s0969-2126(00)00031-9

[15] Uppenberg J., Ohmer N., Norin M., Hult K., Kleywegt G. J., Patkar S., Waagen V., Anthomen T., and Jones T. A. 1995 Crystallographic and molecular-modeling studies of lipase B from Candida antarctica reveal a stereospecificity pocket for secondary alcohols. Biochemistry. 34: 16838–16851. 8527460 10.1021/bi00051a035

[16] Martinelle M., Holmquist M., and Hult K. 1995 On the interfacial activation of Candida antarctica lipase A and B as compared with Humicola lanuginosa lipase. Biochim. Biophys. Acta. 1258: 272–276. 7548197 10.1016/0005-2760(95)00131-u

[17] Trodler P., and Pleiss J. 2008 Modeling structure and flexibility of Candida antarctica lipase B in organic solvents. BMC Struct. Biol. 8: 9. 18254946 10.1186/1472-6807-8-9 PMC2262892

[18] Skjøt M., De Maria L., Chatterjee R., Svendsen A., Patkar S. A., Østergaard P. R., and Brask J. 2009 Understanding the plasticity of the alpha/beta hydrolase fold: lid swapping on the Candida antarctica lipase B results in chimeras with interesting biocatalytic properties. ChemBioChem. 10: 520–527. 19156649 10.1002/cbic.200800668

[19] Ganjalikhany M. R., Ranjbar B., Taghavi A. H., and Moghadam T. T. 2012 Functional motions of Candida antarctica lipase B: a survey through open-close conformations. PLoS One. 7: e40327. 22808134 10.1371/journal.pone.0040327 PMC3393743

[20] Ferrario V., Ebert C., Knapic L., Fattor D., Basso A., Spizzo P., and Gardossi L. 2011 Conformational changes of lipases in aqueous media: a comparative computational study and experimental implications. Adv. Synth. Catal. 353: 2466–2480.

[21] Fisher S. J., Wilkinson J., Henchman R. H., and Helliwell J. R. 2009 An evaluation review of the prediction of protonation states in proteins versus crystallographic experiment. Crystallogr. Rev. 15: 231–259.

[22] Fisher S. J., Blakeley M. P., Cianci M., McSweeney S., and Helliwell J. R. 2012 Protonation-state determination in proteins using high-resolution X-ray crystallography: effects of resolution and completeness. Acta Crystallogr. D Biol. Crystallogr. 68: 800–809. 22751665 10.1107/S0907444912012589

[23] Kabsch W. 2010 XDS. Acta Crystallogr. D Biol. Crystallogr. 66: 125–132. 20124692 10.1107/S0907444909047337 PMC2815665

[24] Vagin A., and Teplyakov A. 1997 MOLREP: an automated program for molecular replacement. J. Appl. Crystallogr. 30: 1022–1025.

[25] Murshudov G. N., Vagin A. A., and Dodson E. J. 1997 Refinement of macromolecular structures by the maximum-likelihood method. Acta Crystallogr. D Biol. Crystallogr. 53: 240–255. 15299926 10.1107/S0907444996012255

[26] Emsley P., Lohkamp B., Scott W. G., and Cowtan K. 2010 Features and development of Coot. Acta Crystallogr. D Biol. Crystallogr. 66: 486–501. 20383002 10.1107/S0907444910007493 PMC2852313

[27] Joosten R. P., Long F., Murshudov G. N., and Perrakis A. 2014 The PDB_REDO server for macromolecular structure model optimization. IUCrJ. 1: 213–220. 25075342 10.1107/S2052252514009324 PMC4107921

[28] Winn M. D., Ballard C. C., Cowtan K. D., Dodson E. J., Emsley P., Evans P. R., Keegan R. M., Krissinel E. B., Leslie A. G. W., McCoy A., 2011 Overview of the CCP4 suite and current developments. Acta Crystallogr. D Biol. Crystallogr. 67: 235–242. 21460441 10.1107/S0907444910045749 PMC3069738

[29] Uppenberg J., Patkar S., Bergfors T., and Jones T. A. 1994 Crystallization and preliminary X-ray studies of lipase B from Candida antarctica. J. Mol. Biol. 235: 790–792. 8289302 10.1006/jmbi.1994.1035

[30] Laskowski R. A., MacArthur M. W., Moss D. S., and Thornton J. M. 1993 PROCHECK: a program to check the stereochemical quality of protein structures. J. Appl. Crystallogr. 26: 283–291.

[31] Cruickshank D. W. J. 1999 Remarks about protein structure precision. Acta Crystallogr. D Biol. Crystallogr. 55: 583–601. 10089455 10.1107/s0907444998012645

[32] Prangé T., Schiltz M., Pernot L., Colloc’h N., Longhi S., Bourguet W., and Fourme R. 1998 Exploring hydrophobic sites in proteins with xenon or krypton. Proteins. 30: 61–73. 9443341

[33] Olczak A., Cianci M., Hao Q., Rizkallah P. J., Raftery J., and Helliwell J. R. 2003 S-SWAT (softer single-wavelength anomalous technique): potential in high-throughput protein crystallography. Acta Crystallogr. A. 59: 327–334. 12832811 10.1107/s0108767303009693

[34] Frey P. A., Whitt S. A., and Tobin J. B. 1994 A low-barrier hydrogen bond in the catalytic triad of serine proteases. Science. 264: 1927–1930. 7661899 10.1126/science.7661899

[35] Kuhn P., Knapp M., Soltis S. M., Ganshaw G., Thoene M., and Bott R. 1998 The 0.78 Å structure of a serine protease: Bacillus lentus subtilisin. Biochemistry. 37: 13446–13452. 9753430 10.1021/bi9813983

[36] Altschul S. F., Madden T. L., Schäffer A. A., Zhang J., Zhang Z., Miller W., and Lipman D. J. 1997 Gapped BLAST and PSI-BLAST: a new generation of protein database search programs. Nucleic Acids Res. 25: 3389–3402. 9254694 10.1093/nar/25.17.3389 PMC146917

[37] Derewenda U., Swenson L., Wei Y., Green R., Kobos P. M., Joerger R., Haas M. J., and Derewenda Z. S. 1994 Conformational lability of lipases observed in the absence of an oil-water interface: crystallographic studies of enzymes from the fungi Humicola lanuginosa and Rhizopus delemar. J. Lipid Res. 35: 524–534. 8014587

[38] Schrag J. D., Li Y., Cygler M., Lang D., Burgdorf T., Hecht H-J., Schmid R., Schomburg D., Rydel T. J., Oliver J. D., 1997 The open conformation of a Pseudomonas lipase. Structure. 5: 187–202. 9032074 10.1016/s0969-2126(97)00178-0

[39] Louwrier A., Drtina G. J., and Klibanov A. M. 1996 On the issue of interfacial activation of lipase in nonaqueous media. Biotechnol. Bioeng. 50: 1–5. 18626893 10.1002/(SICI)1097-0290(19960405)50:1<1::AID-BIT1>3.0.CO;2-L

[40] Laskowski R. A. 2001 PDBsum: summaries and analyses of PDB structures. Nucleic Acids Res. 29: 221–222. 11125097 10.1093/nar/29.1.221 PMC29784

[41] DeLano W. L. 2002. The PyMOL User’s Manual. DeLano Scientific, San Carlos, CA.

[42] McNicholas S., Potterton E., Wilson K. S., and Noble M. E. 2011 Presenting your structures: the CCP4mg molecular-graphics software. Acta Crystallogr. D Biol. Crystallogr. 67: 386–394. 21460457 10.1107/S0907444911007281 PMC3069754

